# The Effects of Anemia on Pregnancy Outcome in Patients with Pyelonephritis

**DOI:** 10.1155/2013/780960

**Published:** 2013-11-27

**Authors:** Sarah K. Dotters-Katz, Chad A. Grotegut, R. Phillips Heine

**Affiliations:** Division of Maternal-Fetal Medicine, Department of Obstetrics and Gynecology, Duke University, DUMD Box 3967, Durham, NC 27710, USA

## Abstract

*Objective*. Pyelonephritis is a common infectious morbidity of pregnancy. Though anemia is commonly associated with pyelonephritis, there are little data describing the effect of pyelonephritis with anemia on pregnancy outcomes. The purpose of this study was to further assess the association of anemia with infectious morbidity and pregnancy complications among women with pyelonephritis. *Study Design*. We conducted a retrospective cohort study of pregnant women admitted to Duke University Hospital between July 2006 and May 2012 with pyelonephritis. Demographic, laboratory, and clinical data from the subject's pregnancy and hospitalizations were analyzed. Patients with pyelonephritis and anemia (a hematocrit < 32) were compared to those without anemia. Descriptive statistics were used to compare the two groups. *Results*. 114 pregnant women were admitted with pyelonephritis and 45 (39.5%) had anemia on admission. There was no significant difference in age, race, preexisting medical conditions, or urine bacterial species between patients with anemia and those without. Women with anemia were more likely to deliver preterm (OR 3.3 (95% CI 1.07, 11.4), *P* = 0.04). When controlling for race and history of preterm delivery, women with anemia continued to have increased odds of preterm birth (OR 6.0, CI 1.4, 35, *P* = 0.012). *Conclusion*. Women with pyelonephritis and anemia are at increased risk for preterm delivery.

## 1. Introduction

Pyelonephritis is the most common nonobstetric indication for antepartum admissions. Upper urinary tract infection complicates 1%-2% of pregnancies and has the potential to cause severe maternal and fetal morbidity [1–3]. The physiologic changes of pregnancy, including decreased ureteral peristalsis, mechanical compression of the ureters, decreased detrusor tone, and incomplete bladder emptying, may predispose pregnant woman to pyelonephritis.

Anemia is a commonly described complication of pyelonephritis [1–6]. The physiology of this association is poorly understood. Confusing the picture is the fact that anemia itself is a common complication of pregnancy and is associated with preterm delivery and small for gestational age infants [7, 8]. Theories regarding the etiology of pyelonephritis-associated anemia include overhydration at diagnosis, endotoxin mediated hemolysis, renal erythropoietin suppression, and anemia of chronic disease [4, 9–11]. Despite the known association of pyelonephritis with anemia, there are minimal data describing the effects of anemia related to pyelonephritis on pregnancy outcomes.

This study was initiated to evaluate pregnancies complicated by pyelonephritis and to explore differences in infectious morbidity and pregnancy complications among pregnant women with pyelonephritis who present with anemia compared to those without anemia.

## 2. Materials and Methods

We conducted a retrospective cohort study of all pregnant patients admitted to the Duke University Hospital from July 2006 to May 2012 with pyelonephritis. Subjects were initially identified by discharge International Classification of Diseases, 9th revision, clinical modification (ICD-9-CM) codes of 646.63 (genitourinary infection in pregnancy, antepartum condition, or complication), 590.80 (pyelonephritis), 590.81 (pyelitis or pyelonephritis), and 590 (kidney infection). From this initial set of subjects, we then identified those who were pregnant with a singleton gestation at the time of diagnosis and who met our definition of pyelonephritis. Pyelonephritis was defined as evidence of urinary tract infection via urinalysis (>8–12 white blood cell count and <2–4 epithelial cells) or urine culture (>100,000 colony forming units) as well as clinical findings of at least one of the following: fever (temperature ≥ 100.4°F), flank pain, or costovertebral angle tenderness [12]. The most common reasons for exclusion were postpartum diagnosis, urinary tract infection without meeting diagnosis of pyelonephritis, and other genitourinary pathology not consistent with pyelonephritis.

Demographic data and clinical and laboratory characteristics from the first prenatal visit, the admission for pyelonephritis, and delivery were collected and analyzed. Hospital admission data for patients who were admitted more than once with a diagnosis of pyelonephritis were only included from the initial admission. However, it was noted that they had a second admission within the data set. Anemia was defined as a hematocrit less than 32% [13]. Leukocytosis was defined as a white blood cell count greater than or equal to 10 × 10^9^. Microcytosis was defined as a mean corpuscular volume of less than 80 fL. Though our institution does not have a strict protocol for treatment of pyelonephritis, all patients in the study were managed in the hospital as an inpatient until afebrile for more than 24 hours on intravenous antibiotics and no longer have pain. Ceftriaxone is the most common primary antibiotic used for treatment of pyelonephritis at our institution (106/114). Other antibiotics used included Ampicillin (*n* = 3), Gentamycin (*n* = 2), Cefazolin (*n* = 1), Aztreonam (*n* = 1), and Unasyn (*n* = 1). Preterm delivery was defined as delivery resulting from spontaneous preterm labor prior to 37 weeks of gestation, not medically indicated preterm delivery.

Categorical variables were compared using chi-squared tests and Fisher's exact test. Continuous variables were compared using two sided Student's *t*-tests. Logistic regression analyses were used to compute odds ratios (OR) with 95% confidence intervals for the outcome preterm delivery among women with pyelonephritis and anemia compared to women with pyelonephritis without anemia. A multivariable logistic regression model was then constructed for the outcome preterm delivery, while also including race/ethnicity, history of prior preterm birth, and chronic medical conditions that varied between the two groups with a *P* value ≤ 0.1 (asthma, diabetes, and sickle cell trait). All analyses were performed using JMP (JMP for Macintosh, version 10.0, SAS Institute, Inc., Cary, NC, USA). A *P*-value of <0.05 was considered significant. This study protocol was reviewed and approved by the Duke University Medical Center Institutional Review Board.

## 3. Results

There were 114 pregnant women admitted with pyelonephritis during the study period. Forty-five women (39.5%) had anemia at the time of their admission, while sixty-nine women did not.  Table 1 provides demographic information comparing those women with anemia and those without. Women with anemia at the time of hospitalization for pyelonephritis had a lower BMI (28.6 versus 31.6, *P* = 0.025) compared to women without anemia. There was no difference in maternal age between the two groups. There was a higher percentage of African-American women who were anemic at the hospitalization for pyelonephritis, while Caucasian and Hispanic women were less likely to have anemia. There was no difference in gestational age at first prenatal visit or parity between the two groups. Women with anemia had similar rates of prenatal care both prior to their admission for pyelonephritis and during pregnancy, compared to women without anemia. 

Table 2 depicts medical conditions present in patients with pyelonephritis among women with and without anemia. There were no significant differences in preexisting medical conditions, including nongestational diabetes, tobacco abuse, recreational drug abuse, chronic hypertension, neurologic diseases, and psychiatric disorders between the two groups. However, women with pyelonephritis and anemia were more likely to have sickle cell trait compared to women with pyelonephritis who did not have anemia (*P* = 0.052), though only two patients in our study had this diagnosis. Women with asthma also may have been more likely to be anemic (17.8% versus 7.3%, *P* = 0.09). Genital-urinary diseases, including anomalies, history of pyelonephritis, recurrent urinary tract infections, history of kidney stones, and ureteral stents were common, occurring in 21.9% of all cases of pyelonephritis.

Clinical and laboratory details describing the admissions for pyelonephritis are described in Table 3. The mean hematocrit at admission in the patients with anemia was 29.2% and 34.4% in nonanemic patients, (*P* = 0.009), while the median hematocrit was 30 (range 25–31) in the anemia group and 34 (range 32–41) in the nonanemic group. The MCV at admission was also lower in women with anemia compared to those without (82.7 versus 85.8, *P* = 0.009). The length of stay during the admission for pyelonephritis did not differ between the two groups. The majority of women in either group were admitted during the second trimester. The mean (SD) gestational age at admission was 25 (5.6) weeks for women with anemia and 22.4 (9.1) weeks for those without (*P* = 0.058). There was no difference in white blood cell count, serum creatinine, or urine analysis values at admission between the two groups (Table 3).

*Escherichia coli *(*E. coli*) was the most common urologic pathogen isolated in both groups and was responsible for 69.3% of all infections. There was no difference in the amount of pan-sensitive *E. coli* or ampicillin resistant *E. coli* between the two groups (Table 3). Other pathogens, including *Klebsiella, Proteus,* and *Enterococcus*, occurred in similar frequencies between the two groups.

There was no difference in the amount of nonobstetric imaging received between the two groups. Two women were admitted to the intensive care unit, both of them were anemic. Four patients received blood transfusions, all of them had a normocytic anemia on presentation and none of them were anemic at their first prenatal visit.

Obstetric outcomes are detailed in Table 4. Among all study patients, the preterm birth rate was 13.7%. There were three women who delivered during admission for pyelonephritis, two at term and one preterm. Women with pyelonephritis who also had anemia were more likely to deliver prematurely (OR, 3.3; 95% CI 1.1, 11.4, *P* = 0.04) compared to women with pyelonephritis who did not have anemia. After controlling for race/ethnicity, history of prior preterm birth, asthma, diabetes, and sickle cell trait women with anemia continued to have increased odds of premature delivery (OR 6.3, 95% CI 1.4, 38.6, *P* = 0.0124) compared to women without anemia. There was no difference in the percentage of small for gestational age infants between the two groups.

In the anemia group, there was laboratory data from the first trimester for 23 of 45 women. Of those, only five had anemia in the first trimester. There were 36 patients with a normocytic (MCV > 80) anemia compared to nine with a microcytic (MCV < 80) anemia. There was no difference in preterm deliveries between the two populations (22% in the microcytic group versus 24.2% in the normocytic group, *P* = 0.9). Nearly 80% of patients with microcytic anemia remained anemic at the time of delivery, compared to only 55% of those with a normocytic anemia (*P* = 0.2). One of the five patients with normocytic anemia delivered an infant that was small for gestational age, compared to 10% of those with a microcytic anemia (*P* = 0.52). Patients with microcytic anemia were younger, presented at a later gestational age, and had a lower hematocrit (32.9% versus 35.4%, *P* = 0.17) at their first prenatal visit, though none of these values reached significance. There were no patients in the microcytic group who went to the ICU or who received blood transfusions.

## 4. Discussion

Pyelonephritis is commonly associated with preterm labor; however, there is mixed data regarding what the actual risk is for preterm delivery. In women who have had antepartum pyelonephritis, studies have found between a 6% and 50% rate of preterm delivery [3, 4, 14, 15]. Other studies showed no increased risk [2, 3]. The role of anemia and its association with preterm birth is not clear. In our study, among women with pyelonephritis, anemia was associated with preterm delivery. While anemia at the time of admission for pyelonephritis did not appear to be a marker for more severe disease nor did it affect the hospital course of the pyelonephritis, there was an increased rate of preterm delivery in the anemic population. This relationship remained after controlling for the known risk factors of prior preterm birth history and race. Women who remained anemic at the time of delivery had a 33% preterm delivery rate, 5 times higher than those whose hematocrit normalized.

The etiology of anemia related to pyelonephritis remains poorly understood, despite being frequently identified in retrospective studies and documented in major textbooks [2, 4, 14, 16]. Though often thought to be secondary to aggressive fluid rehydration, this theory is less probable [4]. Another hypothesis poses hemolysis as the responsible etiology. Cox et al. described 18 women who presented with acute pyelonephritis complicated by anemia [10]. All of these women had increased proportions of echinocytes, schistocytes, and spherocytes, changes consistent with hemolysis [10]. Cavenee et al. postulated that perhaps decreased erythropoietin production related to renal dysfunction was responsible for the anemia [9]. However, further study did not find a difference in erythropoietin levels in pregnant patients with pyelonephritis compared to controls at admission or discharge. Additionally, this study did not see any difference in erythropoietin levels at the time of delivery [9].

Several authors have suggested that anemia associated with pyelonephritis is due to anemia of chronic disease, termed “silent renal infection” [17, 18]. This hypothesis suggests that patients with asymptomatic bacteria actually have underlying dysfunction of the renal parenchyma, which leads to the anemia. The inflammatory response created by a subclinical or silent infection may lead to decreased responsiveness of bone marrow to erythropoietin mediated by inflammatory cytokines, specifically interleukin 1 (IL-1) and tumor necrosis factor alpha (TNF-*α*) and thus anemia [19–21]. Inflammation of the lower reproductive tract with significant elevation of cytokines in both vaginal and amniotic fluid has been associated with preterm delivery. Based on the above hypothesis, the finding that anemic pyelonephritis patients have a higher rate of preterm birth may be in accord with the conclusion that chronic inflammation at sites distant from the reproductive tract may be linked with preterm delivery. This is consistent with the association of periodontal disease and preterm birth in which it is postulated that bacteria egress hematologically and create an inflammatory environment in the lower reproductive tract [22–24].

It is also important to note that there is conflicting data in the literature concerning the risk for preterm delivery among women with anemia in general. In a prospective cohort study of 160,700 women in China, the preterm birth rate, defined as a delivery less than 37 weeks, was 4.1% for women with anemia compared to 5.0% among women without anemia [25]. In contrast, among a large cohort of Israeli women (153,396 deliveries), 10.7% of the women with anemia delivered preterm (defined as less than 37 weeks) compared to 9.4% of women without anemia [26]. At this time, further research is needed concerning the role of anemia on preterm delivery. 

The strengths of this study are threefold. First, this study population is from a single center with uniform treatment. Additionally, the classic risk factors associated with both preterm birth and pyelonephritis did not differ between the population with anemia and the population without anemia. The percentages of minority women with and without anemia were nearly identical but were higher than the percentage of Caucasian women. This is concordant with other studies regarding risk factors for pyelonephritis [1, 2, 4, 6]. Finally, risk factors commonly associated with anemia, such as little or no prenatal care, minority race, substance abuse, and psychiatric disorder did not differ between the anemic and nonanemic subjects with pyelonephritis [2, 6, 27].

Despite these strengths, there are weaknesses as well. First, many of the patients did not have prenatal care prior to admission for pyelonephritis, and thus it is impossible to ascertain if these women were anemic in the first trimester and if they had bacteriuria prior to admission. Also, delivery data were not available for all patients. This may have caused bias to the analysis. As a major referral center, patients in preterm labor are transported to our institution, while many patients who labor at term deliver at their local hospital. We tried to overcome this bias by only including patients who were diagnosed with pyelonephritis at our institution. Our study does not account for patients who were treated as outpatients for pyelonephritis. However, at our institution, it is the practice to admit all patients with this infection. It is not standard of care at our institution to draw lactose dehydrogenase (LDH) or haptoglobin for patients with pyelonephritis. Thus, we were unable to assess for hemolysis in this study. In addition, among women with anemia at the time of admission with pyelonephritis, there was no difference in the mean change in hematocrit from admission with pyelonephritis compared to delivery by gestational age of delivery. We acknowledge that we may have been underpowered to determine if a change in hematocrit affected the risk for preterm delivery. Finally, because of our study design, we could not assess causation but only describe an association. 

This study compared pregnant women with pyelonephritis with and without anemia. The most significant finding in our study is the increased risk for preterm delivery in women with anemia and pyelonephritis. Because there is data which links anemia alone to preterm delivery, further study is needed to assess if the relationship between pyelonephritis, anemia, and preterm delivery is causal or merely an association and, if the relationship is causal, whether the mechanism of action is chronic inflammation. Prospective studies looking at pyelonephritis, anemia, and preterm delivery rates in women with asymptomatic bacteriuria may help to answer this query. If chronic inflammation associated with pyelonephritis and anemia is responsible for the adverse pregnancy outcomes, therapeutic interventions both during and prior to pregnancy could be investigated in this specific patient population.

## Figures and Tables

**Table 1:** Patient demographics.

	Pyelonephritis with anemia *n* = 45 (%)	Pyelonephritis without anemia *n* = 69 (%)	*P* value
Race/ethnicity, *n* (%)			0.109
Caucasian	5 (11.1)	18 (26.1)	
African American	24 (53.3)	23 (33.3)	
Hispanic	15 (33.3)	26 (37.7)	
Other	1 (2.2)	2 (2.9)	
Age, years^a^	23.6 ± 5.7	24.5 ± 5.9	0.41
Body mass index, kg/m^2^	28.6 ± 5.4	31.6 ± 8.1	0.025
Multiparous, *n* (%)	31 (68.9)	39 (56.5)	0.18
Prior preterm delivery, *n* (%)	13 (28.9)	11 (15.9)	0.10
Pyelonephritis more than once in pregnancy, *n* (%)	4 (8.9)	2 (2.9)	0.17
Gestational age at first prenatal visit, weeks^a^	16.1 ± 6.3	14.6 ± 7.1	0.32
No prenatal care before pyelonephritis admission, *n* (%)	31 (68.9)	45 (65.2)	0.68
No prenatal care, *n* (%)	8 (17.8)	11 (15.9)	0.80

^a^Values are mean ± SD.

**Table 2:** Preexisting medical conditions.

Condition, *n* (% )	Pyelonephritis with anemia *N* = 45	Pyelonephritis without anemia *N* = 69	*P* value
Asthma	8 (17.8)	5 (7.3)	0.09
Diabetes (nongestational)	2 (4.4)	9 (13.0)	0.11
Sickle cell trait	2 (4.4)	0 (0)	0.052
Substance abuse	8 (17.8)	10 (14.5)	0.64
Hypertension (chronic)	1 (2.2)	4 (5.8)	0.34
Genitourinary disease	11 (24.4)	14 (20.3)	0.60
Tobacco abuse	3 (6.67)	6 (8.7)	0.69
Psychiatric disorder	8 (17.8)	10 (14.5)	0.64

**Table 3:** Pyelonephritis admission data.

	Pyelonephritis with anemia *n* = 45	Pyelonephritis without anemia *n* = 69	*P* value
Length of stay, days	4.0 ± 1.7	4.1 ± 1.7	0.88
Gestational age at admission, weeks	25 ± 5.6	22.4 ± 9.1	0.058
Gestational age at admission by trimester, *n* (%)			0.0003
1st	0 (0)	13 (18.8)	
2nd	33 (73.3)	34 (49.3)	
3rd	12 (26.7)	22 (31.9)	
Laboratory data			
White blood cell count, ×10^9^ ^a^	13.3 ± 4.6	14.1 ± 4.5	0.42
Hematocrit, %^a^	29.2 ± 1.7	34.4 ± 2.0	<0.0001
MCV, fL^a,b^	82.7 ± 5.7	85.8 ± 6.5	0.009
MCV < 80 fl, *n* (%)^b^	9 (20)	9 (13)	0.32
Creatinine, mg/dL	0.69 ± 0.4	0.66 ± 0.2	0.64
Urinary pathogen, *E. coli*, *n* (% )	29/43 (67.4)	50/68 (73.5)	0.49

^a^Values are mean ± SD.

^b^MCV: mean corpuscular volume.

**Table 4:** Obstetric events present at time of delivery.

Condition, *n* (%)	Pyelonephritis with anemia *n* = 40^a^	Pyelonephritis without anemia *n* = 55^a^	*P* value
Mode of delivery			
Spontaneous vaginal delivery	25 (61.0)	28 (50.9)	0.33
Cesarean delivery	13 (31.7)	21 (38.2)	0.51
Operative vaginal delivery	3 (7.3)	6 (10.9)	0.55
Preterm delivery	9 (22)	4 (7.3)	0.04
Small for gestational age	7 (18)	5 (9.3)	0.22
Hematocrit <32 at delivery	24 (60)	9 (16.4)	<0.001
Hematocrit at delivery, %^b^	30.9 ± 2.8	34.8 ± 3.3	<0.001

^a^Delivery data available for 95 subjects.

^b^Values are mean ± SD.

## References

[1] Gilstrap LC, Cunningham FG, Whalley PJ (1981). Acute pyelonephritis in pregnancy: an anterospective study. *Obstetrics and Gynecology*.

[2] Hill JB, Sheffield JS, McIntire DD, Wendel GD (2005). Acute pyelonephritis in pregnancy. *Obstetrics and Gynecology*.

[3] Sharma P, Thapa L (2007). Acute pyelonephritis in pregnancy: a retrospective study. *Australian and New Zealand Journal of Obstetrics & Gynaecology*.

[4] Duff P (1984). Pyelonephritis in pregnancy. *Clinical Obstetrics and Gynecology*.

[5] Gabbe SG (2012). *Obstetrics Normal and Problem Pregnancies*.

[6] Wing DA (1998). Pyelonephritis. *Clinical Obstetrics and Gynecology*.

[7] Scanlon KS, Yip R, Schieve LA, Cogswell ME (2000). High and low hemoglobin levels during pregnancy: differential risks for preterm birth and small for gestational age. *Obstetrics and Gynecology*.

[8] Xiong X, Buekens P, Alexander S, Demianczuk N, Wollast E (2000). Anemia during pregnancy and birth outcome: a meta-analysis. *American Journal of Perinatology*.

[9] Cavenee MR, Cox SM, Mason R, Cunningham FG (1994). Erythropoietin in pregnancies complicated by pyelonephritis. *Obstetrics and Gynecology*.

[10] Cox SM, Shelburne P, Mason R, Guss S, Cunningham FG (1991). Mechanisms of hemolysis and anemia associated with acute antepartum pyelonephritis. *American Journal of Obstetrics and Gynecology*.

[11] Lucas MJ, Cunningham FG (1993). Urinary infection in pregnancy. *Clinical Obstetrics and Gynecology*.

[12] Archabald KL, Friedman A, Raker CA, Anderson BL (2009). Impact of trimester on morbidity of acute pyelonephritis in pregnancy. *American Journal of Obstetrics and Gynecology*.

[13] Reveiz L, Gyte GM, Cuervo LG, Casasbuenas A (2011). Treatments for iron-deficiency anaemia in pregnancy. *Cochrane Database of Systematic Reviews*.

[14] Dawkins JC, Fletcher HM, Rattray CA, Reid M, Gordon-Strachan G (2012). Acute pyelonephritis in pregnancy: a retrospective descriptive hospital based-study. *ISRN Obstetrics and Gynecology*.

[15] Farkash E, Weintraub AY, Sergienko R, Wiznitzer A, Zlotnik A, Sheiner E (2012). Acute antepartum pyelonephritis in pregnancy: a critical analysis of risk factors and outcomes. *European Journal of Obstetrics Gynecology and Reproductive Biology*.

[16] Cunningham FG, Williams JW (2010). *Williams Obstetrics*.

[17] Fairley KF, Bond AG, Adey FD (1966). The site of infection in pregnancy bacteriuria. *The Lancet*.

[18] Turck M, Ronald AR, Petersdorf RG (1968). Relapse and reinfection in chronic bacteriuria. II. The correlation between site of infection and pattern of recurrence in chronic bacteriuria. *The New England Journal of Medicine*.

[19] Means RT, Krantz SB (1992). Progress in understanding the pathogenesis of the anemia of chronic disease. *Blood*.

[20] Means RT, Krantz SB (1993). Inhibition of human erythroid colony-forming units by tumor necrosis factor requires beta interferon. *The Journal of Clinical Investigation*.

[21] Voulgari PV, Kolios G, Papadopoulos GK, Katsaraki A, Seferiadis K, Drosos AA (1999). Role of cytokines in the pathogenesis of anemia of chronic disease in rheumatoid arthritis. *Clinical Immunology*.

[22] Gervasi MT, Romero R, Bracalente G (2012). Midtrimester amniotic fluid concentrations of interleukin-6 and interferon-gamma-inducible protein-10: evidence for heterogeneity of intra-amniotic inflammation and associations with spontaneous early (<32 weeks) and late (>32 weeks) preterm delivery. *Journal of Perinatal Medicine*.

[23] Vrachnis N, Karavolos S, Iliodromiti Z (2012). Review: impact of mediators present in amniotic fluid on preterm labour. *In Vivo*.

[24] Weissenbacher T, Laubender RP, Witkin SS (2013). Diagnostic biomarkers of pro-inflammatory immune-mediated preterm birth. *Archives of Gynecology and Obstetrics*.

[25] Zhang Q, Ananth CV, Li Z, Smulian JC (2009). Maternal anaemia and preterm birth: a prospective cohort study. *International Journal of Epidemiology*.

[26] Levy A, Fraser D, Katz M, Mazor M, Sheiner E (2005). Maternal anemia during pregnancy is an independent risk factor for low birthweight and preterm delivery. *European Journal of Obstetrics Gynecology and Reproductive Biology*.

[27] Jolley JA, Kim S, Wing DA Acute pyelonephritis and associated complications during pregnancy in 2006 in US hospitals. *The Journal of Maternal-Fetal & Neonatal Medicine*.

